# One Health approach and infection control for colistin-resistant Escherichia coli in China

**DOI:** 10.3205/dgkh000590

**Published:** 2025-09-30

**Authors:** Jiancong Wang

**Affiliations:** 1 Chair of Model-Based Environmental Exposure Science, Faculty of Medicine, University of Augsburg, Augsburg, Germany

## Letter to the editor

There is a limited amount of literature available regarding the One Health approach in China, specifically in relation to controlling infections caused by colistin-resistant *Escherichia (E.) c*oli. Antimicrobial resistance is a growing global health concern that not only affects human health but also animal health through the biological food chain cycle. This paper aims to provide a comprehensive overview of the current status of colistin-resistant *E. coli* for food and poultry in China.

As growth promoter, the use of colistin in agriculture has been identified as a key driver of the emergence and spread of colistin-resistant bacteria in food and animals. Evidence shows that a substantial rise in the prevalence of colistin-resistant *E. coli* among food isolates from 2015 to 2017 in China [1]. Liu et al.[2] reported 15% of 523 raw meat samples were tested positive for colistin-resistant *E. coli*. The spread of colistin-resistant bacteria poses a threat to health beyond individual as these bacteria have the potential to spread from person to person, or from animals to humans, and transfer their resistance genes, making them difficult to treat with antibiotics [3]. There is evidence indicating the emergence of a new mobile colistin resistance gene in Enterobacteriaceae in China, which has been attributed to the overuse of antibiotics in both human medicine and agriculture [2], [4]. 

From a One Health perspective, farmers may not be aware of the risks associated with administering colistin to their poultry and the potential for the resistance. Another possibility is that farmers may not have a complete understanding of the implications of antibiotic resistance and the link between the use of antibiotics in agriculture and the development of resistance in both animals and humans [5]. Collaboration and engagement from multiple stakeholders are necessary for improving awareness and education regarding the risks associated with antibiotic use in agriculture [6]. Several strategies, such as improved regulation, education campaigns, and alternative methods to antibiotics in agriculture, may play a vital role in reducing the risk of antibiotic resistance. 

In China, the challenges of conducting active surveillance for colistin-resistant *E. coli* in food and poultry are further complicated by the lack of standardization in testing methods [7]. This issue is particularly prevalent in rural areas, where most of the poultry production takes place. Additionally, the absence of a mandatory reporting system for antimicrobial resistance in food and agriculture further exacerbates the situation. Identifying colistin-resistant *E. coli* is just the beginning; however, the complex supply chain and distribution networks of food and poultry products pose a challenge in tracing the origin and spread of these bacteria. Compounding this issue is the presence of many asymptomatic carriers of resistant strains, making detection and control of their spread difficult. Enhancing active surveillance for antimicrobial resistance in food and poultry is crucial for mitigating the risk of antibiotic resistance.

The successful infection control measures implemented in the Netherlands and Canada to control the use of colistin and reduce the prevalence of colistin-resistant *E. coli* in food and poultry are worth learning from. The Netherlands has implemented a policy of “prudent use” of antibiotics in animal production to reduce antibiotic use in animal husbandry and prevent the development of antibiotic resistance [8]. Additionally, the use of a probiotic feed additive containing lactobacilli has been found to reduce colistin-resistant *E. coli* in broiler chickens and has been implemented as a control measure. Furthermore, Canada’s One Health approach, led by the Canadian Integrated Program for Antimicrobial Resistance Surveillance (CIPARS), monitors antimicrobial resistance in food, animals, and humans. CIPARS has implemented strict regulations and education programs to promote responsible antibiotic use among farmers, veterinarians, and the public. This approach has successfully reduced the prevalence of colistin-resistant *E. coli* in food and poultry through evidence-based policy and decision-making [9].

The first step that China should take in addressing the issue of colistin-resistant *E. coli* in food and poultry is to establish a comprehensive and integrated surveillance system to monitor the prevalence of antimicrobial resistance in the food chain. This should involve regular testing of food products and live animals, as well as environmental samples from farms and processing facilities. Secondly, it should work to reduce the use of antibiotics, including colistin, in agriculture. This can be achieved through the development and implementation of national policies and regulations that promote responsible use of antibiotics, as well as the adoption of alternative strategies such as improved biosecurity measures. Another important step is to increase awareness and education among farmers, veterinarians, and other stakeholders about the risks associated with the use of antibiotics in food and poultry production, and the importance of responsible use. This can be achieved through targeted education and outreach programs, as well as through collaboration with industry associations and other stakeholders. Finally, it should promote a One Health approach to addressing antimicrobial resistance, involving collaboration between human health, animal health, and environmental health sectors. This can help to ensure that interventions are comprehensive, coordinated, and effective in reducing the spread of antimicrobial resistance. Overall, a combination of regulatory, educational, and collaborative approaches will be needed to effectively address the issue of colistin-resistant *E. coli* in food and poultry in China.

## Notes

### Competing interests

The authors declare that they have no competing interests.

### Funding

None.

### Author’s ORCID

- Wang J: https://orcid.org/0000-0001-6284-9702
